# A Comprehensive Evaluation of the Process of Copying a Complex Figure in Early- and Late-Onset Alzheimer Disease: A Quantitative Analysis of Digital Pen Data

**DOI:** 10.2196/18136

**Published:** 2020-08-12

**Authors:** Ko Woon Kim, Sung Yun Lee, Jongdoo Choi, Juhee Chin, Byung Hwa Lee, Duk L Na, Jee Hyun Choi

**Affiliations:** 1 Department of Neurology School of Medicine Jeonbuk National University Hospital Jeonju Republic of Korea; 2 Research Institute of Clinical Medicine of Jeonbuk National University Jeonju Republic of Korea; 3 Biomedical Institute of Jeonbuk National University Hospital Jeonju Republic of Korea; 4 Department of Physics Pohang University of Science and Technology Pohang Republic of Korea; 5 Center for Neuroscience Korea Institute of Science and Technology Seoul Republic of Korea; 6 Department of Neurology Samsung Medical Center Sungkyunkwan University School of Medicine Seoul Republic of Korea; 7 Neuroscience Center Samsung Medical Center Seoul Republic of Korea; 8 Department of Health Sciences and Technology, SAIHST Sungkyunkwan University Seoul Republic of Korea; 9 Stem Cell & Regenerative Medicine Institute Samsung Medical Center Seoul Republic of Korea; 10 Samsung Alzheimer Research Center Samsung Medical Center Seoul Republic of Korea

**Keywords:** alzheimer disease, Rey-Osterrieth Complex Figure, digital biomarkers, copying process

## Abstract

**Background:**

The Rey-Osterrieth Complex Figure Test (RCFT) is a neuropsychological test that is widely used to assess visual memory and visuoconstructional deficits in patients with cognitive impairment, including Alzheimer disease (AD). Patients with AD have an increased tendency for exhibiting extraordinary behaviors in the RCFT for selecting the drawing area, organizing the figure, and deciding the order of images, among other activities. However, the conventional scoring system based on pen and paper has a limited ability to reflect these detailed behaviors.

**Objective:**

This study aims to establish a scoring system that addresses not only the spatial arrangement of the finished drawing but also the drawing process of patients with AD by using digital pen data.

**Methods:**

A digital pen and tablet were used to copy complex figures. The stroke patterns and kinetics of normal controls (NCs) and patients with early-onset AD (EOAD) and late-onset AD (LOAD) were analyzed by comparing the pen tip trajectory, spatial arrangement, and similarity of the finished drawings.

**Results:**

Patients with AD copied the figure in a more fragmented way with a longer pause than NCs (EOAD: *P*=.045; LOAD: *P*=.01). Patients with AD showed an increased tendency to draw the figures closer toward the target image in comparison with the NCs (EOAD: *P*=.005; LOAD: *P*=.01) Patients with AD showed the lower accuracy than NCs (EOAD: *P*=.004; LOAD: *P*=.002). Patients with EOAD and LOAD showed similar but slightly different drawing behaviors, especially in space use and in the initial stage of drawing.

**Conclusions:**

The digitalized complex figure test evaluated copying performance quantitatively and further elucidated the patients’ ongoing process during copying. We believe that this novel approach can be used as a digital biomarker of AD. In addition, the repeatability of the test will delineate the process of executive functions and constructional organization abilities with disease progression.

## Introduction

### Background

The Rey-Osterrieth Complex Figure Test (RCFT) is widely used to examine the visuoconstruction and visual memory function of patients with brain injuries or cognitive impairment such as Alzheimer disease (AD) [1]. The conventional scoring system of RCFT focuses on scoring the final finished drawing of the figure by assessing the shape and positional accuracy of its elements. There are only a few scoring methods that quantify the drawing sequences of elements [2-4]. However, patients with cognitive impairment due to brain injuries show different patterns in the drawing process and errors on the final finished drawing compared with normal controls (NC; ie, individuals with normal cognition) [5]. For example, some patients with brain injuries complete a figure by adding one detailed part after another instead of starting with an overall outline and adding local or detailed parts. Furthermore, the number, length, and speed of strokes made by patients with brain injuries may differ from that of NCs. Therefore, it is necessary to establish a scoring system using a digital device that records not only the spatial arrangement of the finished drawing but also all the details of drawing processes, including the sequence in drawing the parts of the figures. This approach will augment our understanding of the organizing strategy and executive functions of patients with brain injuries. Additionally, it will increase our understanding of the structural and design integrity of drawing figures.

A digital pen and tablet can be optimal tools for observing and acquiring data for the drawing process. Recent studies have used a digital device to evaluate the visuoconstructional abilities and executive functions of patients with AD. These researchers used a clock drawing test [6,7] or a 3D house copy test [8] to quantitatively evaluate cognitive function in patients with AD. It was possible to differentiate patients with mild cognitive impairment (MCI) and AD from NCs by assessing physical parameters such as kinetics (eg, time, velocity) or device-human interaction values (eg, pressure, number/density of strokes). Compared with NCs, even patients with MCI who received normal scores on the clock drawing test showed prolonged transition times associated with drawing performances [6]. The kinetics and sequence-based schemes from digital drawing tests have successfully demonstrated changes in cognitive progress. Previously, only 1 study has analyzed RCFT using a digital device; however, the group did not assess the drawing procedure but focused on recognizing the outline and details in relation to the conventional scoring system [9].

### Objective

To implement the scheme in a digital device, we first decided to choose an abstract figure such as the RCFT rather than concrete objects such as a clock or a house. Clocks and houses elicit semantic knowledge, which primarily involves the ventral visual pathway (*what* pathway). Using complex figures on the other hand, such as the RCFT, involves the dorsal visual pathway (*where* pathway) and requires visuospatial working memory before translation into a motor program and execution of plan [10]. Second, we simplified the Rey figure to reduce not only the drawing time but also the number of strokes required to complete the figure. Arranging too many structures in a limited space may result in stroke overlap when drawing, which may prevent the digital device from identifying each stroke.

In this study, we hypothesized that the movement kinetics (pen tip trajectory of the digital pen) and digitized spatial information acquired from the simplified RCFT would differ between NCs and patients with AD. Several previous studies have reported that patients with early-onset AD (EOAD) have significant visuospatial or visuoconstructive difficulties compared with patients with late-onset AD (LOAD) [11-13]. We therefore hypothesized that patients with EOAD would show more pronounced changes in movement kinetics and digitized spatial information compared with patients with LOAD.

## Methods

### Recruitment of Participants

Participants were selected from those who visited the Memory Disorders Clinic at the Samsung Medical Center in Seoul, Republic of Korea, between March 1 and December 1, 2017. The study group comprised 17 patients with EOAD, 21 patients with LOAD, and 17 NC individuals. We consecutively recruited participants who satisfied the following criteria: (1) normal visual acuity, (2) >6 years of education, (3) completion of a standardized neuropsychological battery called the Seoul Neuropsychological Screening Battery (SNSB) [14,15] and the Mini-Mental State Examination (MMSE) test [16], and (4) magnetic resonance imaging (MRI) results. Participants were classified via rigorous diagnostic methods, including multiple tests (eg, blood test, MRI/positron emission tomography scans) and clinical consensus of neurologists, neuropsychologists, and radiologists. All patients with AD fulfilled the criteria proposed by the National Institute of Neurological and Communicative Disorders and Stroke and the Alzheimer’s Disease and Related Disorders Association (NINCDS-ADRDA) [17]. Patients with EOAD were defined as those whose first symptoms occurred at an early age (>45 years and <65 years of age). Individuals with moderate or severe vision loss (visual acuity <0.3) or those with very low MMSE scores with lower cutoffs of 10 or education levels <6th grade were not included in this study.

### Neuropsychological Assessments

All participants underwent a standardized neuropsychological battery called the SNSB [14,15], which consists of tests for attention, language, calculation, visuospatial, memory, and frontal/executive functions. The MMSE and Clinical Dementia Rating tests were also carried out to evaluate general cognition. The standardized tests used for this study were as follows: attention was assessed using the backward digit span and letter cancellation tests; language was assessed using the Korean version of the Boston Naming Test; calculation was assessed using 3 items comprising each for addition, subtraction, multiplication, and division; visuospatial function was assessed using the RCFT; memory function was assessed using immediate and delayed recall of the Seoul Verbal Learning Test and RCFT; frontal-executive function was assessed using the phonemic and semantic Controlled Oral Word Association Test and the Stroop word/color reading test. Each score was converted into a standardized Z-score based on age- and education-adjusted norms.

### Simplified Version of the Rey-Osterrieth Complex Figure Test

**Figure 1:**
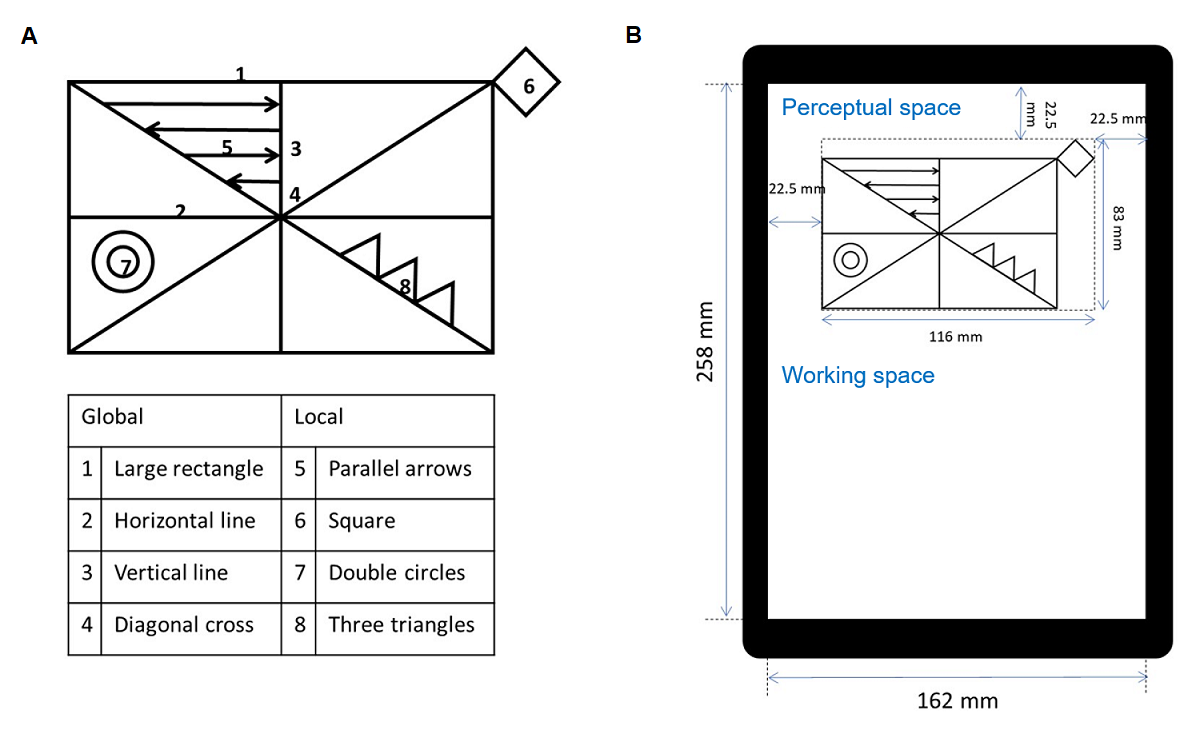
The simplified Rey-Osterrieth Complex Figure Test (RCFT). (A) The simplified RCFT consists of 4 global and 4 local components. (B) The simplified RCFT is shown on a tablet (size: 12 in; resolution: 2160 × 1440; Samsung Galaxy Book 12, Samsung Electronics) with a screen width of 162 mm and a screen height of 258 mm. The texts indicated in blue were not presented to the participants during testing.

We modified the original RCFT into a simpler version while maintaining the main frame of the figure. Modification was performed by 1 neurologist (experience >10 years) and 2 neuropsychologists (average experience >20 years) to balance the numbers of global and local components. Although the original RCFT comprises 18 components (4 global and 14 local components) [18], our simplified RCFT comprised 4 global and 4 local components, as illustrated in Figure 1. The large rectangle with horizontal, vertical, and diagonal crosses was maintained, but other regional or local features were simplified except for the 4 horizontal lines in the upper left panel. For the local features, the components in the original RCFT were changed as follows: (1) the diamond was replaced by a square, (2) the circle with 3 dots was replaced by double circles, (3) the 5 parallel lines were replaced by 3 triangles, (4) and the 4 parallel lines were replaced by 4 arrows. We added arrows to these lines such that the participants would be more attentive to the local features. Some overlapping lines and detailed components outside of the outlines were eliminated in the simplified version. For the global components, the side of the large triangle attached to the large rectangle and the horizontal line of the large triangle were excluded. Of the local components, the following were removed: the vertical cross, the small triangle above the large rectangle, the horizontal cross, the square attached to the large rectangle, the small rectangle, the small horizontal line above the small rectangle, the vertical line within the side of the large triangle, and the small vertical line within the large rectangle.

Each of the 8 components in the simplified RCFT was scored separately (Meyers and Meyers protocol) in terms of accuracy and placement [18]. Component scores were assigned as 2 (accurately drawn, correctly placed), 1 (accurately drawn, incorrectly placed or inaccurately drawn, correctly placed), 0.5 (inaccurately drawn, incorrectly placed but recognizable), or 0 (inaccurately drawn, incorrectly placed, unrecognizable). Thus, the possible range of raw scores was 0.0-16.0. A low score suggested impaired visuoperceptual or visuoconstructive functions.

### Experimental Apparatus (Digitized Equipment) and Drawing Procedure

The experimental equipment for measuring the performance of copying a figure consisted of an X-Y digital tablet (size: 12-inch, resolution: 2160 × 1440; Samsung Galaxy Book 12, Samsung Electronics) and a digital pen (nib diameter: 0.7 mm, pressure: 4096; S-pen, Samsung Electronics). Participants performed the simplified RCFT on a tablet in portrait orientation (Figure 1). We defined the upper half of the working area as *perceptual space* and the lower half as *working space*. The simplified RCFT was presented in the perceptual space. An eraser tool was not included in the interface to observe the basal level of copying. Before the task, the experimenter delivered the following instructions to the participants: “When the task begins, a figure will appear on the upper half of this tablet. Please copy the figure with this pen. Try to use the empty area below as much as possible. This pen does not have an eraser. You cannot change your drawing. If you made any mistakes, please disregard them and continue with carrying out the task. Take your time, and please let me know when you are finished*.*” The trajectories of the drawings were recorded on the X-Y digital tablet at a sampling frequency of 60 Hz, and the x- and y-axes were horizontal and vertical planes, respectively.

### Data Processing in Normalized Cartesian Coordinates

The digital pen-touch point responses were recorded by the X-Y digital tablet as Cartesian coordinates in pixel units based on placement on the screen, movement, and removal from the screen over time. The range of positions was (0, 0) to (2160, 1440), which corresponded to the range of display in pixels. We normalized the position from a range of –1 to 1 with respect to the origin (0, 0) at the center of the display. The vertical ranges of perceptual and working spaces corresponded to 0 to 1 and –1 to 0, respectively. To consider the robustness of the automatic evaluation, we focused on analyzing parameters such as pen stroke, occupied drawing area (drawing boundary), and copying results (similarity between the original figure).

We analyzed 3 features: (1) digital pen stroke, which was defined as continuous movement of the digital pen while maintaining contact with the writing tablet; (2) drawing boundary, defined as the extremum coordinates; and (3) copying results (similarity of the drawing to the original). These features were selected based on their robustness to automatic evaluation.

#### Analysis of Pen Strokes

**Figure 2:**
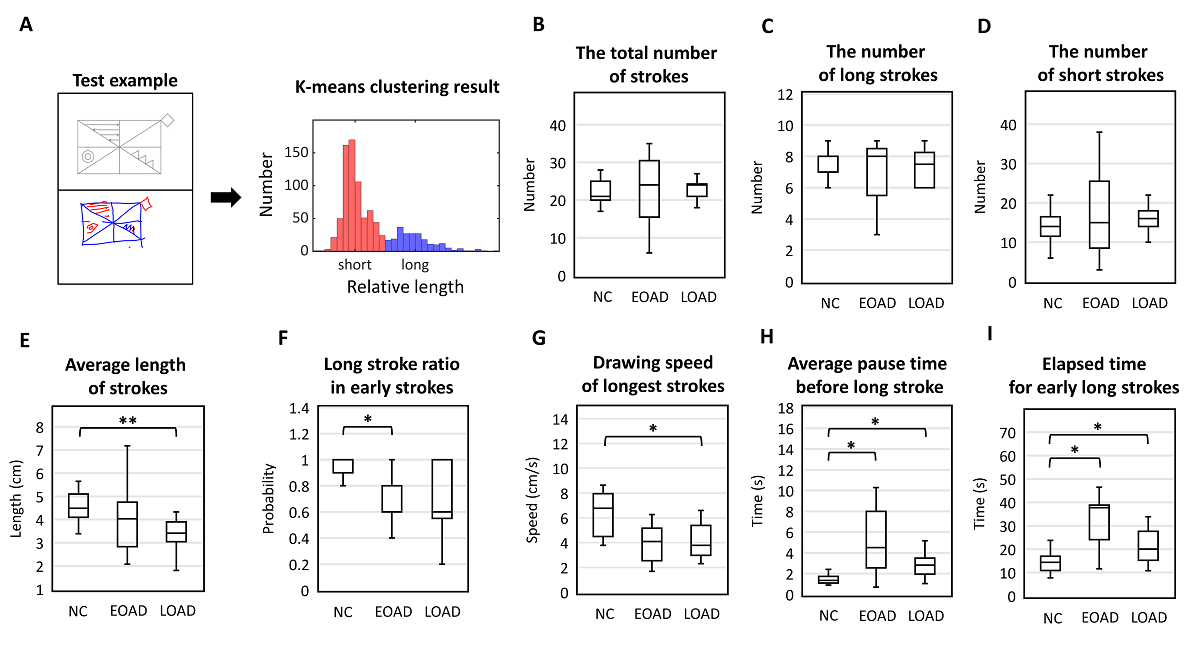
Pen stroke analysis. Clinical interpretation of copying performance in the Rey-Osterrieth Complex Figure Test (RCFT) was partly based on how an individual draws the image based on consecutive line creations. The number, length, and speed of strokes were analyzed. (A) Long (blue)/short (red) strokes are classified by k-means clustering; (B-D) number of strokes; (E-F) length of strokes; (G-I) speed of strokes. One and two asterisks show significant differences at *P*<.05 and *P*<.01 levels, respectively. EOAD: early-onset Alzheimer disease; LOAD: late-onset Alzheimer disease; NC: normal control.

Pen stroke was referred to as a trajectory created based on continuous contact of the digital pen. Specifically, pen stroke was defined as pen movements recorded from the moment the pen touched the screen to the moment the pen was lifted off the screen. To analyze stroke behaviors, the number, length, and speed were assessed. We also analyzed data after classifying strokes as long or short lines (Figure 2).

#### Analysis of Drawing Areas (Spatial Arrangement of Drawings)

**Figure 3:**
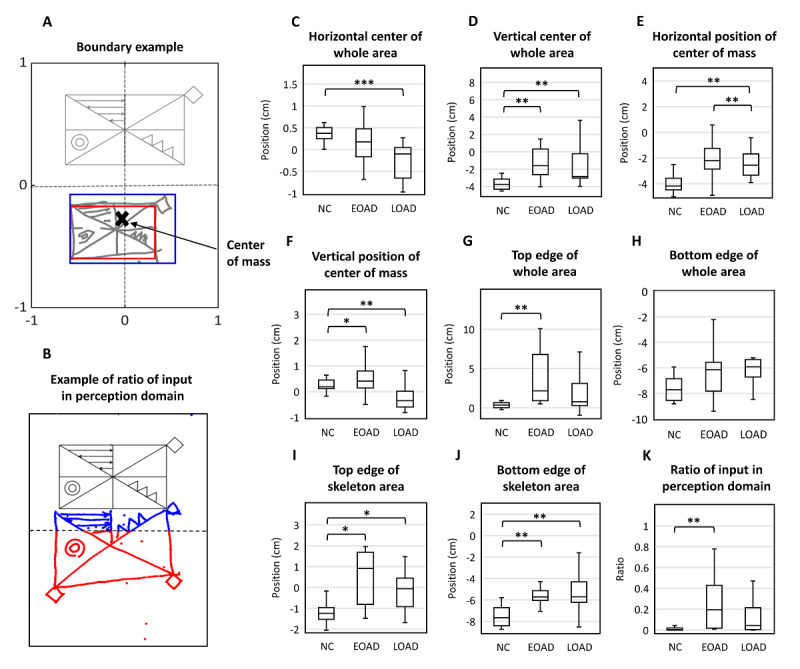
Spatial arrangement. (A) We defined the bounded area that contains the whole drawing as the whole area (blue box) and a boundary that contains only the area inside the skeleton rectangle as the skeleton area (red box). (B) An example of the ratio of input in perceptual space: input (blue)/total input (blue + red). (C-D) Center of the whole area. (E-F) Center of mass of the whole area. (G-H) Top and bottom edge of the whole area. (I-J) Top and bottom edges of the skeletal area. (K) Closing-in phenomenon. One, two, and three asterisks show significant differences at *P*<.05, *P*<.01, and *P*<.001 levels, respectively. EOAD: early-onset Alzheimer disease; LOAD: late-onset Alzheimer disease; NC: normal control; RCFT: Rey-Osterrieth Complex Figure Test.


To evaluate the space occupancy of drawings and possible signs of unilateral neglect of space, we defined the boundary of the drawing region and measured its area and lateralization. To compare the space occupancy for all items with that of the global items (large square with diagonal axes), we defined 2 boundaries of *whole area*, which contained the whole drawing, and *skeleton area*, of that within the rectangle border (Figure 3). For ease of calculation, we defined the area as a square. The entire area included all input drawings and was determined by 4 extremums along the horizontal and vertical axes. By definition, the skeleton area was the boundary of the square created by minimizing the area following consideration of all global features. To perform automatic calculations, we determined the skeleton area using the following steps: (1) the input frequency was determined at each subregion in which the working space was divided into 10×10 subregions, (2) inputs in subregions with frequencies lower than 1 out of 4 were excluded, and (3) the extremums were obtained with the remaining inputs and defined as the skeleton area. After detection of boundaries, we obtained the center, vertex, and edge lengths of the regions. We further calculated the center of mass, 
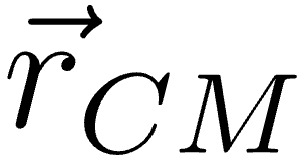
, using the following equation:

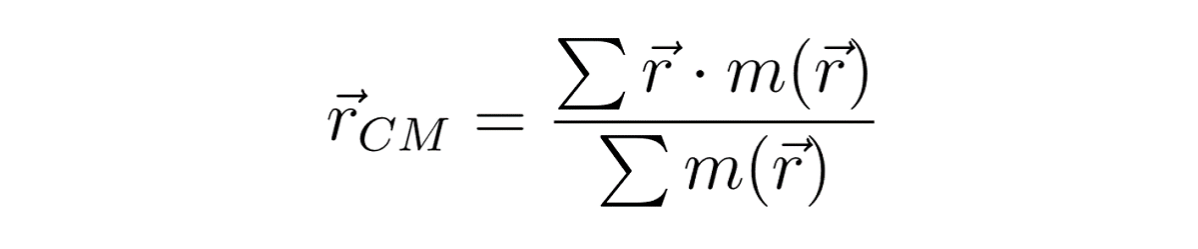
 **(1)**

where m=1 if there is input at the ith pixel at or 0 if not.

#### Analysis of Copying Similarity

**Figure 4:**
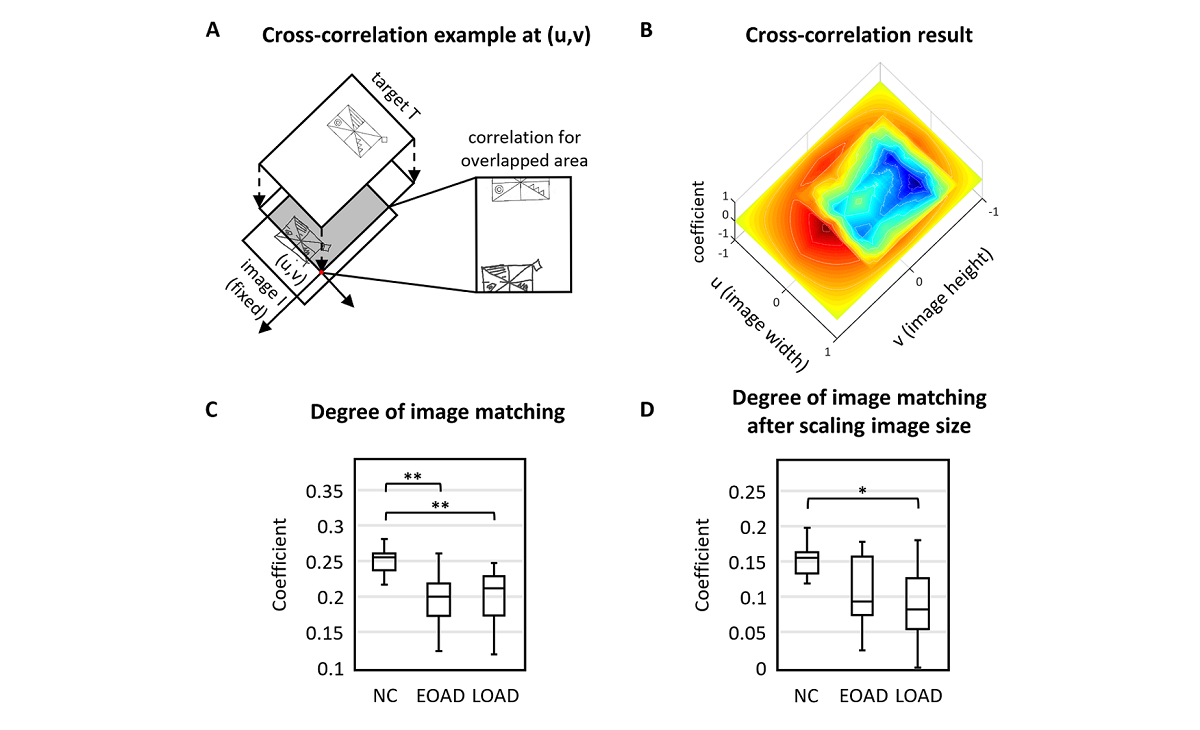
Shape similarity between the target and the pictures drawn by the participants. (A) The Pearson correlation coefficient was used to assess cross-correlation for all possible overlap. (B). We applied 2D cross-correlation analysis and calculated its coefficient and shift in space. (C) For both AD groups, pattern matching was significantly poorer than that of the normal control (NC) group (pairwise Mann-Whitney U test: *P*=.004 for early-onset Alzheimer disease [EOAD] vs NCs and *P*=.002 for late-onset Alzheimer disease [LOAD] vs NCs). There was no statistical significance between the EOAD and LOAD groups (*P*=.86). (D) Although the amount of difference was reduced, the similarity was significantly lower for the LOAD group even after rescaling the image size (*P*=.04). There was no statistical significance for the EOAD group even after adjusting for image size. One and two asterisks show significant differences at *P*<.05 and *P*<.01 levels, respectively. RCFT: Rey-Osterrieth Complex Figure Test.

To evaluate the accuracy of copying, we performed a 2-dimensional (2D) cross-correlation analysis Figure 4. In this analysis, the input function *m* was scanned over the perceptual and working spaces with respect to the stimulus function *M*, with a value of 0 or 1 in the absence or presence of input, respectively. The amplitude distribution of the 2D cross-correlation function, 
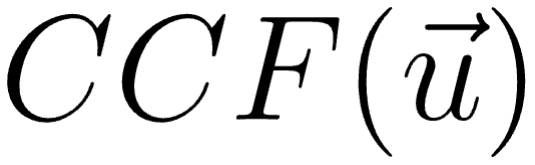
, was calculated as:


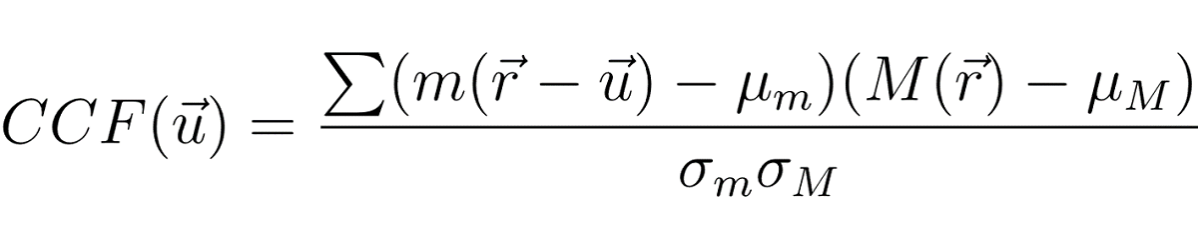
 **(2)**

where μ_M_ and μ_m_ were the means of stimulus and input signals, respectively. The SD was computed as follows:


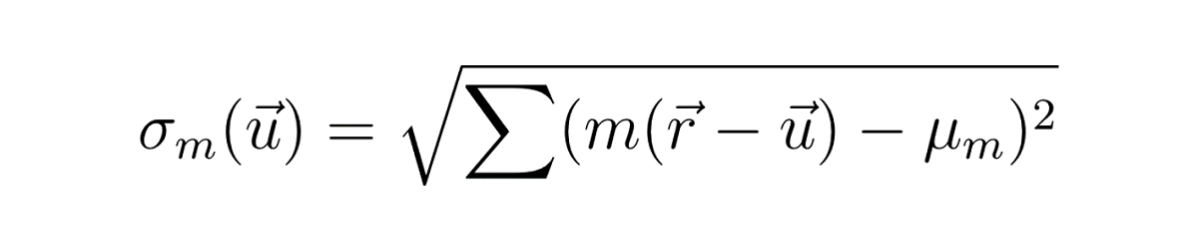
 **(3)**

for σ_m_and equivalently for σ_M_ without 
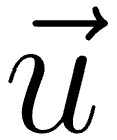
. All summation was performed within the overlapped area between *m* and *M*, when *m* was located at 
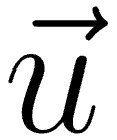
. The normalized cross-correlation function had values between −1 and +1. The maximum value of 
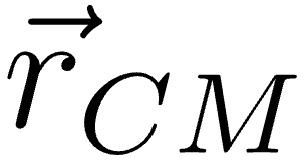
 represented the similarity between the 2 patterns. Due to variation in size of the whole area between the images, we calculated the maximum correlation after rescaling along the x-axis. The coefficient of 
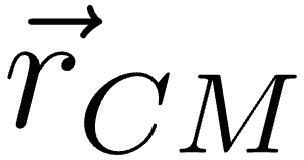
 was obtained by determining its peak, which was assumed to be the center of the attention field.

### Statistical Analysis

For statistical analysis of the demographic and cognitive profile data, the Kolmogorov-Smirnov test was used to conduct normality tests. The Kruskal-Wallis test was used to examine the statistical significance between groups at a significance level of *P*=.05 because variables did not follow a normal distribution. We performed post hoc comparisons using the pairwise Mann-Whitney U test with Bonferroni correction. We used the chi-square test or Fisher exact test for categorical variables, followed by a Bonferroni post hoc analysis. To validate the simplified Rey figures, we used Pearson correlation to compare conventional RCFT scores and simplified RCFT scores.

For statistical analysis of digital pen data, the Lilliefors test was used to assess the normality and the Kruskal-Wallis test was again used to examine the statistical significance between groups at a confidence level of *P*=.05. We performed post hoc comparisons using the pairwise Mann-Whitney U test with a Dunn-Šidák correction by adjusting the *P* value of each pairwise test as p=1–(1–p_o_)^3^, where 3 reflects the number of groups. We compared the immediate/recall/recognition scores of the original RCFT and our digital metrics from the simplified RCFT using Pearson correlation to determine whether there are any data linked to memory function. All hypothesis tests were two-tailed. MATLAB R2018b was used for the calculations.

## Results

### Demographics and Cognitive Profiles

The demographic and cognitive profiles of the participants are summarized in Table 1. Age (*P*<.001) and education (*P*=.10) differed significantly among NCs and participants with EOAD and LOAD. The prevalence of apolipoprotein E4 carriers among NCs (0/13, 0%) was significantly lower than that among patients with EOAD (10/16, 63%) or LOAD (9/20, 45%). To investigate differences in neuropsychological scores among the 3 groups, we used Z scores based on age- and education-adjusted norms. Both patients with EOAD and LOAD showed poor performance in attention, language, visuospatial function, memory, and frontal/executive functions compared with NCs (Table 1).

Before the analysis, digital data were reviewed carefully, and data that were not appropriate were excluded from the analysis. A total of 3 participants were excluded because of mismatched digital pen data between attach/detach and movement pairs; 8 participants were excluded because of missing attach movements in coordinate data; 6 participants were excluded because of mismatched digital pen data between result image and pen movement. Digital data from a total of 11 NCs, 11 patients with EOAD, and 16 patients with LOAD were analyzed.

**Table 1:** Clinical characteristics.

Demographics and cognitive profiles		NC^a^ (n=17)	EOAD^b^ (n=17)	LOAD^c^ (n=21)	Post hoc Bonferroni test (*P* value)		
					NC vs EOAD	NC vs LOAD	EOAD vs LOAD
Age (years), n (IQR)		73 (65.5-77.0)	65 (58.5-68.0)	78 (74.0-81.5)	*.011* ^d^	*.005*	*<.001*
**Gender**							
	Female:male	8:9	8:9	8:13	>.99	*<.001*	>.99
Education (years), n (IQR)		16 (10.5-16.0)	12 (9.0-13.0)	12 (11.0-16.0)	.12	.36	>.99
APOE4^e^ carrier^f^, n (%)		0^g^ (0)	10^h^/16 (63)	9^i^/20 (45)	*.001*	*.015*	>.99
Amyloid PET^j^ positive^k^, n		0	15	8	*.004*	*.018*	>.99
**Attention, span (IQR)**							
	Forward digit span	0.06 (–0.43 to 1.08)	0.12 (–1.50 to 20.99)	–0.15 (–0.59 to 0.83)	.86	>.99	>.99
	Backward digit span	–0.16 (–0.55 to 1.19)	–0.82 (–1.66 to –0.03)	–0.13 (–1.07 to 0.80)	*.04*	>.99	0.41
**Language, score (IQR)**							
	K-BNT^l^	0.27 (–1.90 to 0.86)	–0.92 (–2.52 to –0.08)	–2.60 (–3.38 to –1.61)	*.002*	*<.001*	0.15
	Calculation	12 (12-12)	10 (7-12)	10 (8-12)	*.003*	*.001*	>.99
**Visuospatial function, score (IQR)**							
	RCFT^m^: copying	0.55 (–0.70 to 0.93)	–1.72 (–7.61 to 0.00)	–1.02 (–7.78 to 0.12)	*0.008*	*0.001*	>.99
**Memory, score (IQR)**					*<.001*	*<.001*	
	SVLT^n^: immediate recall	0.83 (0.24-1.48)	–2.25 (–2.85 to –0.95)	–1.79 (–2.45 to –0.87)			>.99
	SVLT: delayed recall	0.88 (0.34-1.22)	–2.96 (–3.18 to –2.53)	–2.51 (–2.74 to –2.09)			*.008*
	SVLT: recognition	0.89 (0.61-1.10)	–2.23 (–3.93 to –1.94)	–2.66 (–3.26 to –1.51)			>.99
	RCFT: immediate recall	0.86 (0.61-1.10)	–2.23 (–3.93 to –1.94)	–2.66 (–3.26 to –1.51)			>.99
	RCFT: delayed recall	1.36 (0.05-1.76)	–2.11 (–2.55 to –1.82)	–2.17 (–2.45 to –1.65)			>.99
	RCFT: recognition	1.01 (0.04-1.47)	–2.48 (–2.75 to –2.16)	–2.36 (–2.58 to –1.97)			.56
**Frontal/executive functions, score (IQR)**							
	COWAT^o^ animal	0.23 (–0.30 to 0.98)	–2.21 (–3.14 to –1.89)	–2.36 (–2.96 to –1.17)	*<.001*	*<.001*	>.99
	COWAT supermarket	0.23 (–0.78 to 1.00)	–1.60 (–2.06 to –1.32)	–1.73 (–1.99 to –1.04)	*.001*	*.001*	>.99
	COWAT phonemic	0.70 (0.00-1.59)	–1.37 (–1.61 to –0.47)	–0.85 (–1.68 to 0.28)	*<.001*	*.01*	>.99
	Stroop test: color	0.56 (–0.17 to 1.03)	–3.22 (–3.89 to –0.75)	–1.93 (–2.86 to –0.48)	*<.001*	*<.001*	.66
	MMSE^p^	29 (28-30)	22 (15-25)	20 (17-22)	*<.001*	*<.001*	>.99
	CDR^q^	0.5 (0.5-0.5)	1.0 (0.5-1.0)	1.0 (0.75-1.0)	*<.001*	*<.001*	>.99
	CDR sum of box	0.5 (0.5-0.75)	5.0 (4.25-6.25)	4.5 (4.0-8.5)	*<.001*	*<.001*	>.99
	GDS^r^	1.0 (0.0-4.0)	2.0 (1.0-5.0)	1.0 (0.5-7.0)	>.99	.71	>.99

^a^NC: normal control.

^b^EOAD: early-onset Alzheimer disease.

^c^LOAD: late-onset Alzheimer disease.

^d^Italicization show significant differences at *P*<.05.

^e^APOE4: apolipoprotein E4.

^f^APOE4 was analyzed in 49 patients: 13 NCs, 16 patients with EOAD, and 20 patients with LOAD. Participants with one or more copies of the ε4 allele (ie, ε2/4, ε3/4, ε4/4) were considered as ε4 carriers.

^g^n=13.

^h^n=16.

^i^n=20.

^j^PET: positron emission tomography.

^k^Amyloid PET was analyzed in 26 patients: 3 NCs, 15 patients with EOAD, and 8 patients with LOAD. Amyloid PET positivity was interpreted based on previously reported guidelines for each ligand.

^l^K-BNT: Korean version of the Boston naming test.

^m^RCFT: Rey-Osterrieth Complex Figure Test.

^n^SVLT: Seoul Verbal Learning Test.

^o^COWAT: Controlled Oral Word Association Test.

^p^MMSE: Mini-Mental State Examination.

^q^CDR: clinical dementia rating.

^r^GDS: geriatric depression scale.

### Comparison Between Original and Simplified Rey-Osterrieth Complex Figure Test Scores

**Figure 5:**
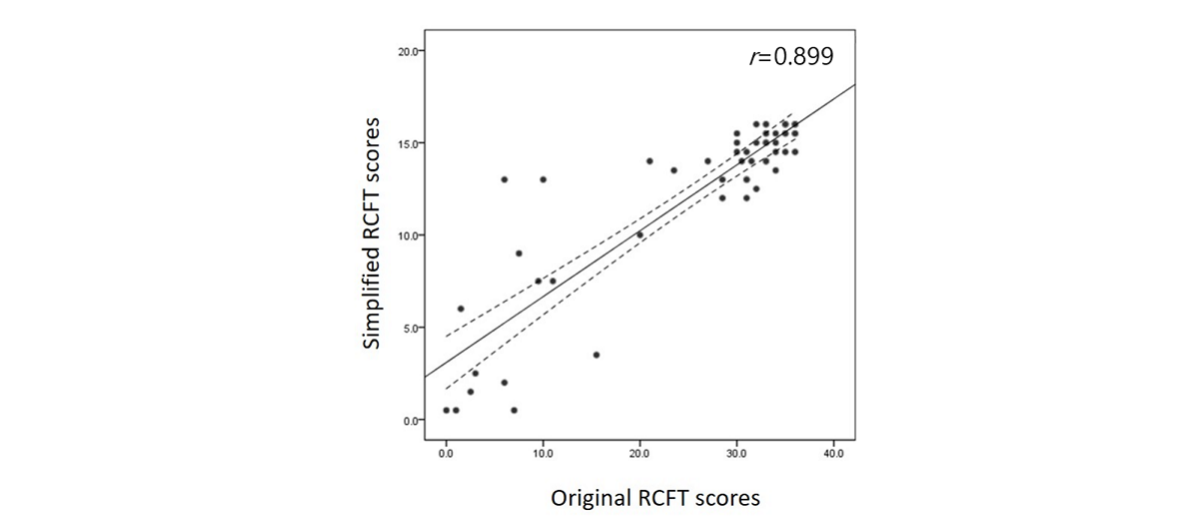
Correlation between original and simplified Rey-Osterrieth Complex Figure Test (RCFT) scores. All participants performed both original and simplified RCFTs. The simplified RCFT was manually scored separately in terms of both accuracy and placement, and it complied with the Meyers and Meyers’ standardized scoring of the original RCFT. Raw scores ranging from 0.0 to 16.0 can be obtained. There was a significant linear relationship between the conventional and simplified RCFT scores (r=0.889; *P*<.001).

Table 2 shows the median and interquartile range raw copy scores of the original and simplified RCFT results from NCs, patients with EOAD, and patients with LOAD. The NCs showed significantly higher scores than the 2 AD groups in both the simplified (EOAD: *P*<.001; LOAD: *P*<.001) and original RCFT tests (EOAD: *P*<.001; LOAD: *P*<.001). There was no significant difference between patients with EOAD and patients with LOAD in the simplified or original RCFT. There was a significant linear relationship between the conventional RCFT and the simplified RCFT score (r=0.884; *P*<.001; Figure 5).

**Table 2:** Raw copy scores for the original and simplified Rey-Osterrieth Complex Figure Tests.

Types of copy scores	NC^a^ (n=17)	EOAD^b^ (n=17)	LOAD^c^ (n=21)	Post hoc Bonferroni test (*P* value)		
				NC vs EOAD	NC vs LOAD	EOAD vs LOAD
Simplified RCFT^d^ (0, 16; IQR)	15.5 (15-16)	12.5 (3.8-14.4)	13 (7.8-15)	*<.001* ^e^	*<.001*	.69
Original RCFT (0, 36; IQR)	35 (33-35)	28 (10-32)	30 (8-31)	*<.001*	*<.001*	>.99

^a^NC: normal control.

^b^EOAD: early-onset Alzheimer disease.

^c^LOAD: late-onset Alzheimer disease.

^d^RCFT: Rey-Osterrieth Complex Figure Test.

^e^Italicization show significant differences at *P*<.001.

### Comparison Between Original Rey-Osterrieth Complex Figure Test Scores and Digital Metrics From Simplified Rey-Osterrieth Complex Figure Test

We compared the immediate/recall/recognition scores of the original RCFT and the digital data acquired from our simplified RCFT to examine whether any of our digital data could be associated with memory function using Pearson correlation. A positive correlation (*r*=0.353; *P*=.04) was observed between immediate recall scores of the original RCFT and the coefficients of shape similarity. In addition, immediate recall scores of the original RCFT and vertical positions of the simplified RCFT on the y-axis negatively correlated as follows: vertical position of the center of the whole area (*r*=–0.341; *P*=.045), vertical position of the center of the skeletal area (*r*=–0.336; *P*=.048), vertical position of the center of the mass (*r*=–0.339; *P*=.047), vertical position of the top edge of the whole area (*r*=–0.343; *P*=.04), and location of the top edge of the skeleton area (*r*=–0.350; *P*=.04). However, there were no significant correlations between recall/recognition scores of the original RCFT and the digital data from the simplified RCFT.

### Analysis of Copying Sequences

**Figure 6:**
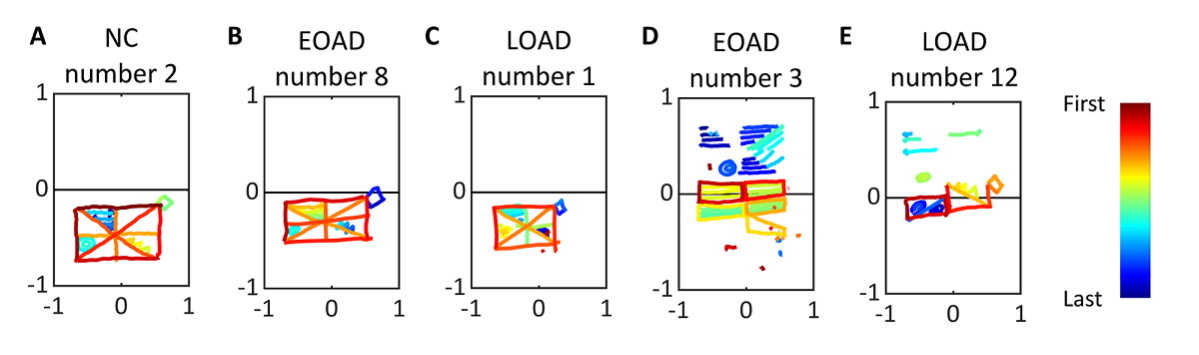
Representative pseudocolored drawings for each group. Pseudocolored drawings of (A) normal control (NC) individuals, (B) patients with early-onset Alzheimer disease (EOAD), and (C) patients with late-onset Alzheimer disease (LOAD), who drew all the elements. (D) Patients with EOAD and (E) LOAD who failed to reproduce the stimulus image. The strokes were colored based on the order of the drawings.

The organizing abilities of the participants were evaluated by analyzing the copying sequences. We converted the original drawing of the simplified Rey figure to pseudocolored images coded by a series of colors in the order of strokes (Multimedia Appendices 1-3). Specifically, the total number of strokes made by each participant was broken down into segments according to the order of strokes, and each segment was coded with a series of colors ranging from red to blue. For instance, when an individual made 40 strokes in total, the first 4 strokes that accounted for the first 10% (4/40) were assigned a red color and the next 10% (4/40) as yellow and so on. Most of the participants drew the global features first and then added the local features, as exemplified in Figure 6. However, some patients started drawing local features such as the double circle or the triangles, as shown in the representative figures (Figure 6) during the middle of drawing global features. In addition, 1 patient with EOAD (number 5 in Multimedia Appendix 2) completed the global structure last. Nonetheless, the tendency of drawing global elements in the beginning was preserved across all subjects. In the unorganized copy as well, participants started with the large box (Figure 6) and then moved onto the local elements.

### Pen Stroke Analysis

Clinical interpretation of copying performance in the RCFT was partly based on individual performance in copying. Therefore, we assessed the number, length, and speed of sequential line strokes (Table 3).

**Table 3:** Digital data of simplified Rey-Osterrieth Complex Figure Tests.

Type of digital data analysis		NC^a^ (n=11)	EOAD^b^ (n=11)	LOAD^c^ (n=16)	Post hoc Dunn-Šidák test (*P* value)		
					NC vs EOAD	NC vs LOAD	LOAD vs EOAD
**Pen stroke analysis, mean (SD)**							
	Total number of strokes	24 (7)	26 (16)	23 (4)	>.99	>.99	>.99
	Number of long strokes	14 (4)	18 (12)	16 (4)	>.99	>.99	>.99
	Number of short strokes	8 (1)	9 (7)	7 (3)	>.99	>.99	>.99
	Length of strokes (cm)	4.58 (0.71)	3.99 (1.48)	3.50 (0.86)	.34	*.007* ^d^	.90
	First 5 stroke ratios	0.91 (0.16)	0.69 (0.23)	0.68 (0.28)	.04	.07	>.99
	Speed of the longest stroke (cm/s)	6.31 (1.86)	4.41 (2.76)	4.08 (1.41)	.09	*.01*	>.99
	Transition time (second)	1.39 (0.60)	2.95 (1.83)	6.23 (7.54)	*.045*	*.01*	.97
	Elapsed time of 5 early long strokes (second)	14.43 (4.62)	34.86 (19.39)	23.65 (14.16)	*.012*	*.04*	.32
**Spatial arrangement, mean (SD)**							
	Whole area (cm^2^)	84.17 (10.88)	108.51 (58.81)	84.01 (33.93)	>.99	>.99	>.99
	Skeleton area (cm^2^)	58.40 (8.79)	56.34 (20.78)	55.29 (18.93)	>.99	>.99	>.99
	Horizontal center of the whole area	0.42 (0.31)	0.25 (0.67)	–0.23 (0.40)	.60	*<.001*	.11
	Vertical center of the whole area	–3.70 (0.69)	–1.22 (1.84)	–1.61 (2.36)	*.005*	*.01*	.79
	Horizontal position of center of mass	0.28 (0.34)	0.46 (0.64)	–0.35 (0.59)	.74	*.004*	*.01*
	Vertical position of center of mass	–3.96 (0.83)	–2.05 (1.68)	1.90 (2.12)	*.02*	*.007*	.99
	Top edge of the whole area	0.25 (0.62)	–3.98 (3.59)	2.37 (3.23)	*.004*	.19	.24
	Bottom edge of the whole area	–7.64 (1.00)	–6.44 (1.97)	–5.59 (1.85)	.21	*.005*	.80
	Top edge of the skeleton area	–1.14 (0.62)	0.89 (2.45)	1.10 (3.46)	*.03*	*.04*	.95
	Bottom edge of the skeleton area	–7.64 (1.00)	–6.44 (1.97)	–5.59 (1.85)	*.008*	*.005*	>.99
	Ratio of input	0.01 (0.02)	0.25 (0.26)	0.18 (0.27)	*.005*	.12	.61
**Shape similarity, mean (SD)**							
	Cross-correlation maximum value	0.25 (0.02)	0.19 (0.05)	0.20 (0.04)	*.004*	*.002*	.86
	Size-rescaled cross-correlation maximum value	0.14 (0.04)	0.11 (0.05)	0.09 (0.05)	.38	*.04*	.67

^a^NC: normal control.

^b^EOAD: early-onset Alzheimer disease.

^c^LOAD: late-onset Alzheimer disease.

^d^Italicization show significant differences at *P*<.05.

#### Number of Pen Strokes

The average number of strokes was 24 (SD 7) in NC individuals, 26 (SD 16) in patients with EOAD, and 23 (SD 4) in patients with LOAD. The total number of strokes did not differ across groups (Figure 2; Kruskal-Wallis test: *P*=.56).

#### Length of Pen Strokes

We used a union of 2 separate stroke sets with x- and y-projected lengths. The average length of the strokes was 4.58 (SD 0.71) cm for NCs, 3.99 (SD 1.48) cm for patients with EOAD, and 3.50 (SD 0.86) cm for patients with LOAD. The average length of strokes was significantly shorter in patients with LOAD than NC individuals (Figure 2; pairwise Mann-Whitney U test: *P*=.007). Extensive variation in the length of long strokes was noted in patients with EOAD.

When drawing figures, the length of strokes may be related to the constructional strategy. Longer strokes are more likely to be associated with a focus on global structure, whereas shorter lines are more likely to be associated with a focus on local features. Thus, the strokes were operationally defined as long or short using a 1-dimensional k-means++ algorithm (Figure 2). The average centroids of each long and short stroke cluster were 1.30 cm and 8.85 cm, respectively, for the x-projected length and 0.858 cm and 5.34 cm, respectively, for the y-projected length. The average ratios between the longest short stroke and the shortest long stroke were 2.20 (SD 0.60) cm for the x-projected length and 1.73 (SD 0.42) cm for the y-projected length. The average number of long strokes was 14 (SD 4) for NCs, 18 (SD 12) for patients with EOAD, and 16 (SD 4) for patients with LOAD. The average number of short strokes was 8 (SD 1) for NCs, 9 (SD 7) for patients with EOAD, and 7 (SD 3) for patients with LOAD. The number of long or short strokes did not differ across the 3 groups (Figure 2; Kruskal-Wallis test: *P*=.58 for long strokes and *P*=.57 for short strokes). The first 5 strokes were defined as the beginning of the task. Most long strokes were drawn at the beginning of the task, but the ratio was significantly lower in patients with EOAD compared with that of NC individuals (Figure 2; pairwise Mann-Whitney U test: *P*=.04).

#### Speed of Pen Strokes

The longest line was used to calculate the drawing speed because it is most likely used to construct the skeleton of the figure, which is indicative of executive function. The average speed of the longest stroke was 6.31 (SD 1.86) cm/s for NCs, 4.41 (SD 2.76) cm/s for patients with EOAD, and 4.08 (SD 1.41) cm/s for patients with LOAD. Patients with LOAD were significantly slower in drawing longer lines compared with NC individuals (Figure 2; pairwise Mann-Whitney U test: *P*=.01).

When completing the task, we noticed that some patients with AD were hesitant in drawing global components. Thus, we first calculated the transition time for drawing a long line after a short line, followed by the elapsed time for the early long strokes. Transition time was defined as the time to initiate a long stroke after drawing a short stroke in all short-long sequences. The average pause time was 19.69 (SD 60.71) seconds for NCs, 14.03 (SD 26.67) seconds for patients with EOAD, and 25.35 (SD 83.81) seconds for patients with LOAD. Excluding the outlier over 60 seconds, the average pause time was 1.3 (SD 0.60) seconds for NCs, 6.23 (SD 7.54) seconds for patients with EOAD, and 2.95 (SD 1.83) seconds for patients with LOAD. Both patients with EOAD and LOAD took longer to initiate long strokes compared with NCs (Figure 2; pairwise Mann-Whitney U test: *P*=.045 for NC vs EOAD: *P*=.01 for NC vs LOAD), whereas the NC individuals showed a relatively consistent pause time. Second, the elapsed time was calculated for 5 early long strokes. Usually, it takes 5 strokes to draw the square and horizontal/vertical/diagonal lines inside the square. The average elapsed time of the 5 strokes was significantly longer for both patients with AD compared with the NC individuals (14.43, SD 4.62 seconds for NCs; 34.86, SD 19.39 seconds for patients with EOAD; and 23.65, SD 14.16 seconds for patients with LOAD). In addition, both patients with AD spent more time drawing global features compared with the NC participants (Figure 2; pairwise Mann-Whitney U test: *P*=.01 for NC vs EOAD: *P*=.04 for NC vs LOAD). None of the parameters stated above differed between patients with EOAD and LOAD.

### Spatial Arrangement

#### Drawing Area

The working space or the drawing area may reflect the spatial arrangement abilities of the participants. We created a boundary that contained the whole drawing and defined it as the *whole area*, and another boundary that contained only the area inside the skeleton rectangle was defined as the *skeleton area* (Figure 3). The size and location of the whole area and skeletal area were analyzed for each group (Table 3). The average whole area was 84.17 (SD 10.88) cm^2^ for the NCs, 108.51 (SD 58.81) cm^2^ for patients with EOAD, and 84.01 (SD 33.93) cm^2^ for patients with LOAD. The average skeleton area was 58.40 (SD 8.79) cm^2^ for NCs, 56.34 (SD 20.78) cm^2^ for patients with EOAD, and 55.29 (SD 18.93) cm^2^ for patients with LOAD. The whole and skeletal areas did not differ among the 3 groups (Kruskal-Wallis test: *P*=.485 for the external boundary and *P*=.36 for the internal boundary). Significant differences were also not noted in the ratios between the whole and skeleton areas (Figure 3; Kruskal-Wallis test: *P*=.21).

#### Center of the Whole Area

Negative values represent a leftward or downward deviation from the center of the working space, whereas positive values represent a rightward or upward deviation from the center of the working space (Table 3). The average horizontal position of the center of the whole area was 0.42 (SD 0.31) cm for NCs, 0.25 (SD 0.67) cm for patients with EOAD, and –0.23 (SD 0.40) cm for patients with LOAD. For the horizontal position, patients with LOAD showed a significant left bias compared with NCs (Figure 3; pairwise Mann-Whitney U test: *P*<.001). The average vertical position of the center of the whole area was –3.70 (SD 0.69) cm for NCs, –1.22 (SD 1.84) cm for patients with EOAD, and –1.61 (SD 2.36) cm for patients with LOAD. For the vertical position, both AD groups showed a significant upward bias compared with NCs (Figure 3; pairwise Mann-Whitney U test: *P*=.005 for NC vs EOAD and *P*=.01 for NC vs LOAD). The position of the center of the skeleton area did not differ among the 3 groups.

#### Center of Mass of the Inputs

We analyzed the center of mass to determine whether the local features were skewed toward 1 side of the drawing (Figure 3). Again, negative numbers denoted leftward and downward deviations, whereas positive numbers indicated rightward and upward deviations from the center of the working space (Table 3). The average horizontal position of the center of mass was 0.28 (SD 0.34) cm for NCs, 0.46 (SD 0.64) cm for patients with EOAD, and –0.35 (SD 0.59) cm for patients with LOAD. With respect to the center of mass in the horizontal dimension, a significant left bias in patients with LOAD was observed compared with NCs and patients with EOAD (Figure 3; pairwise Mann-Whitney U test: *P*=.004 for LOAD vs NCs, *P*=.01 for LOAD vs EOAD). The average vertical position of the center of mass was –3.96 (SD 0.83) cm for NCs, –2.05 (SD 1.68) cm for patients with EOAD, and –1.90 (SD 2.12) cm for patients with LOAD. Compared with the NC individuals, the center of mass in the vertical direction was higher for both AD groups (Figure 3; pairwise Mann-Whitney U test: *P*=.02 for NC vs EOAD and *P*=.007 for NC vs LOAD).

#### Edge of the Whole and Skeleton Area

The invasion of the drawing into the perceptual space was assessed by analyzing the whole area of the top edge (Figure 3; Table 3). The average top edge of the whole area was 0.25 (SD 0.62) cm for NCs, –3.98 (SD 3.59) cm for patients with EOAD, and 2.37 (SD 3.23) cm for patients with LOAD. Negative numbers denoted downward deviations, whereas positive numbers indicated upward deviations from the center of the working space. The patients with EOAD presented a significantly higher value of invasion compared with the NCs, whereas the difference between patients with LOAD and NC was not significant (Figure 3; pairwise Mann-Whitney U test: *P*=.004 for NC vs EOAD and *P*=.19 for NC vs LOAD). Regarding the bottom edge of the whole area, the patients with LOAD showed a significant upward bias compared with NCs, whereas the difference between patients with EOAD and NCs was not significant (Figure 3; pairwise Mann-Whitney U test: *P*=.21 for NC vs EOAD and *P*=.005 for NC vs LOAD).

The average top edge of the skeleton area was –1.14 (SD 0.62) cm for NCs, 0.89 (SD 2.45) cm for patients with EOAD, and 1.10 (SD 3.46) cm for patients with LOAD. Both AD groups showed a significantly higher value of invasion toward the perceptual space compared with that of NC individuals (Figure 3; pairwise Mann-Whitney U test: *P*=.03 for NC vs EOAD, *P*=.045 for NC vs LOAD). In addition, for the bottom edge of the skeleton area, both AD groups showed a significant upward bias compared with NC individuals (Figure 3; pairwise Mann-Whitney U test: *P*=.008 for NC vs EOAD and *P*=.005 for NC vs LOAD).

#### Closing-In Phenomenon

The closing-in phenomenon is the tendency to draw near or on the target when copying the image and is common in patients with AD or vascular dementia [19]. An upward deviation from the center and edge of the whole area may also represent the closing-in phenomenon. To achieve greater accuracy, we assessed *the ratio of input* in the perceptual space to measure the distance the drawing invaded into the perceptual space (Figure 3). Here, the term *the ratio of input* refers to the ratio of data points in the perceptual space to the total data points drawn by the participants. Figure 3 shows an example of the ratio of input in the perceptual space. Patients with EOAD (0.25, SD 0.26) showed a significant amount of invasion compared with NCs (0.01, SD 0.02), whereas the difference between patients with LOAD (0.18, SD 0.27) and NCs (0.01, SD 0.02) was not significant (Figure 3; pairwise Mann-Whitney U test: *P*=.005 for NC vs EOAD and *P*=.12 for NC vs LOAD; Table 3).

### Shape Similarities Between the Target and Drawings Made by Participants

To scale the similarity between the original and copied figures, we applied a 2D cross-correlation analysis and calculated its coefficients and shifts in space. Although the conventional rating evaluates the accuracy of the shape and spatial arrangement of parts, the coefficient signifies the overall similarity between the 2 images (Table 3). To check the validity of this new similarity coefficient, we used Pearson correlation to compare the visual rating scores of the simplified RCFT and the similarity coefficients. According to the analysis, the coefficients of the shape similarity positively correlated to the visual rating (*r*=0.779; *P*<.001).

For both AD groups, the coefficient representing pattern matching was significantly lower than that of NC individuals (pairwise Mann-Whitney U test: *P*=.004 for EOAD vs NCs and *P*=.002 for LOAD vs NCs; Figure 4). However, there was no statistically significant difference between patients with EOAD and patients with LOAD (*P*=.86). Compared with the target stimulus, several participants drew images that were larger or smaller in size; thus, we normalized the copied figures and then performed a 2D cross-coefficient analysis. We found that the similarity was significantly lower in the patients with LOAD compared with that of the NC individuals even after rescaling the image sizes (*P*=.04). However, the significance of the difference between the patients with EOAD and the NC individuals disappeared after adjusting for image size (Figure 4).

## Discussion

### Principal Findings

This study introduced a novel analytic method that uses a digital pen and tablet to evaluate a completed drawing and movement kinetics involved in the drawing process to compare the visuoconstructional abilities of patients with AD and NC individuals. First, we created a simplified RCFT and compared the scores of the simplified Rey figure with those of the original to validate our simplified RCFT. We then compared the 2 AD groups with the NC individuals in terms of the simplified RCFT-derived digital metrics that were analyzed based on 3 aspects: (1) the number, speed, length, and sequence of pen strokes; (2) the spatial arrangement of the drawing; and (3) the similarity between the target picture and the drawing of the participants. Significant differences were noted between the NC and the 2 AD groups. Taken together, our findings suggest that movement kinetics in the drawing process and scores acquired from the finished drawings can serve as useful biomarkers to investigate visuoconstructional dysfunction in AD [20].

### Validation of Simplified Rey-Osterrieth Complex Figure Test

We created a simplified RCFT to reduce drawing time and the number of strokes required to complete the picture. In the limited space, reducing the number of strokes makes it easier for the strokes to be identified by the digital device. Compared with the original, a high correlation in the simplified RCFT score was observed in both the whole and the separate groups (NC and the 2 AD groups; Figure 5; Table 2). In line with previous studies that reported strong correlations between simpler and original RCFT in normal adults [21] and patients with AD [22], our findings suggest that the simplified and original RCFTs may be comparable in evaluating visuoconstructional dysfunction in patients with AD. It was interesting to note that there were outliers among those with lower original RCFT scores: scores of 0 to 10 on the original yielded various scores of 1 to 14 on the simplified version (Figure 5). This could have occurred because patients with substantial visuospatial impairment may become overwhelmed when encountering a complex figure so that they eventually become less motivated to complete a figure. However, the same individuals would be relatively more willing to complete a figure if it was less complicated. These findings may also have implications that our simplified figure may be more appropriate for individuals with mild to moderate than severe impairment of cognition or visuospatial abilities.

### Analysis of Pen Stroke Data and Copying Sequences

Regarding the digital pen stroke data analysis, we first compared the 2 AD groups (EOAD and LOAD) with the NC individuals in terms of temporal appearance of long stokes. Overall, the AD groups used fewer long strokes than the NC group. In addition, both the AD groups drew long strokes later in the drawing process compared with that of the NC group. This suggested that the AD groups tended to draw local features earlier and used shorter strokes to finish a figure than the NC individuals. A previous study reported that more than half of the NC individuals first drew the global features of RCFT and then the details or local features subsequently [23]. Other studies suggest that piecemeal approaches in drawing are indicative of brain disease and that they also reflect the inability of an individual to process global information equivalent to that of NC individuals [18].

Additionally, our digital device enabled us to track the sequences of the drawing by color coding the strokes. In the pencil and paper version of the RCFT, neuropsychologists often track copying sequences by giving the patients a series of color pencils. For example, the red color pencil for the first 10 seconds and the yellow pencil for the next 10 seconds, and so on [2]. Similarly, in our digitized images, we assigned colors to the strokes in a certain order. We found that all NC individuals maintained a similar sequence of drawing, starting with the large box and then drawing the diagonal or orthogonal axes. Most patients who successfully copied all the elements also followed the sequence of the NC individuals. Unconventional approaches to drawing, such as drawing details before finishing the global structure or completing the global structure at the end, were identified from some of the patients. Such behavior, however, was not characterized as unimodal features but rather as a portrayal of an individual’s drawing process. Color coding strokes following the completion of a test may be advantageous over using color pencils because switching color pencils will interrupt the drawing process and thus affect the overall memory processing of the participants.

The first and second methods employed in the pen stroke analysis, as described earlier, suggest that analysis using a digital device can allow quantification of the global versus the local nature of drawing in patients with AD. Regarding the anatomical substrates of global and local processing, it is known that the left hemisphere is more specialized in processing local rather than global features, and vice versa for the right hemisphere. This construct, however, is not consistent with our findings as typical AD involves the bilateral temporoparietal area in a symmetrical fashion. Rather, the *global element first and local element later* strategy may be related to the organizing abilities of individuals that can be mediated by prefrontal cortices, which is one of the brain regions affected in the early stage of AD. Another explanation could be the visual perception hypothesis. Previous studies have reported that the visual perception of normal individuals is a sequential process in which global features are perceived before local features. Another study also had patients with AD read Navon figures (large *global* digits composed of smaller *local* digits) and found that patients showed impairment in reading global figures [24]. Therefore, the preference for drawing local features from the AD groups in our study may indicate a mild form of simultanagnosia, which is part of the Balint syndrome.

Another feature we noticed from our pen stroke analysis was that not only the average length of strokes was significantly shorter in patients with LOAD compared with NC individuals, but the average speed of the longest stroke was also significantly slower in patients with LOAD compared with NC individuals (Figure 2). Age-related changes could have contributed to the results because the older the group was, the longer it took for the participants to complete a stroke (shortest to longest in time: EOAD<NC<LOAD). Statistically significant differences were only identified when comparing the NC and LOAD groups. However, we observed another movement kinetics finding that was inconsistent with our age account. Both patients with EOAD and patients with LOAD had longer pause and elapsed times to initiate long strokes compared with the time that NC individuals took (Figure 2). Therefore, decreased length/speed and increased transition and pause time might be related to Parkinsonism or psychomotor speed slowing in both groups of patients with AD. Unfortunately, we did not evaluate the Unified Parkinson’s Disease Rating Scale (UPDRS) score. Therefore, although we excluded patients with Parkinsonism or degenerative disorders that accompany Parkinsonism, such as progressive supranuclear palsy or Lewy body dementia, while enrolling participants, we cannot eliminate the possibility of the Parkinsonism factored in our data.

### Spatial Arrangement and Closing-In Phenomenon

On the basis of our analysis of the position of the drawing area, patients with AD tended to draw the figures closer toward the perception space containing the target figure. This upward deviation is indicative of the closing-in phenomenon. Previous studies have analyzed copied figures of the alternating square and triangle task and showed that as patients drew from left to right, the drawing approached the target, resulting in a sloping image. The angle of the slope was steeper in patients with AD and vascular dementia in comparison with that of NCs [25,26]. Other studies used the RCFT to analyze the closing-in phenomenon but relied on visual rating rather than quantification [27,28]. To the best of our knowledge, this is the first study to quantify the closing-in phenomenon using the RCFT. We analyzed the x and y coordinates of the drawing area and the input data ratio of the drawing to quantify the closing-in phenomenon (Figure 3). We have successfully demonstrated the difference between patients with AD and normal individuals. The underlying mechanisms of the closing-in phenomenon have not been fully elucidated. Previous studies have explained that the closing-in phenomenon is a part of constructional apraxia or primitive behaviors [26]. Another study analyzed the severity of closing-in as a function of figure complexity and suggested that the closing-in phenomenon is related to the patients’ compensatory strategies to overcome visuospatial dysfunction or visuospatial working memory deficits [19,29,30]. The higher immediate recall scores of the original RCFT associated with higher vertical positions of the simplified RCFT observed in this study also strongly support that the closing-in phenomenon is related to the visuospatial working memory deficit. Overall, the results of this study provide supporting evidence that using a digital device enables quantification of the closing-in phenomenon.

### Shape Similarities

Our 2D cross-correlation analysis calculated a coefficient reflecting the overall similarity between the target picture and the drawings of the participants. The coefficient of both AD groups was significantly lower compared with that of the NCs, although the significance of the difference between the patients with EOAD and the NC individuals disappeared after adjusting the image size. Furthermore, there was a significant correlation between the similarity coefficients and the visual rating scores of the simplified RCFT. Therefore, automatic evaluation of similarity indices, which is not possible when using pen and paper, is another advantage of our analytic method that involves a digital device.

### Early-Onset Alzheimer Disease Versus Late-Onset Alzheimer Disease

Contrary to our expectations, there was no significant difference between patients with LOAD and patients with EOAD, except that unlike patients with LOAD, patients with EOAD showed signs of leftward deviation when drawing the entire figure. Clinically, visuospatial dysfunction and apraxia are more prominent in patients with EOAD than in patients with LOAD [11,12]. A previous study also showed that 33% of patients with EOAD showed nonmemory symptoms, whereas only 6% of the patients with LOAD showed these symptoms [12]. These nonmemory symptoms included language and executive and visuoconstructional functions [31]. Another study that used RCFT to assess visuoconstructional abilities showed that the EOAD group was significantly more impaired than the LOAD group [11]. In contrast to these previous studies, there was no difference between the EOAD and the LOAD groups when analyzing various visuospatial parameters, except the horizontal position of the entire figure. The large variation in the EOAD group could have accounted for the lack of statistical significance.

### Representing Cognitive Domains and Limitation

Thus far, we have addressed 3 aspects of digital metrics derived from the simplified RCFT. It is known that there are several components in human visuospatial function such as visuoperceptual, visuoconstructional, or visuospatial working memory abilities [30,32,33]. Therefore, it may be important to discuss which visuospatial abilities are related to our digital metrics. First, pen stroke data may be linked to motor skills and strategies for the construction of visual components [33]. Second, analysis of the position of the drawing area may be related to the closing-in phenomenon, which reflects visuoperceptual or visuospatial working memory deficits [30]. Third, a 2D cross-correlation analysis may be associated with general visuospatial abilities that include both perception and construction attributes of visuospatial function [32]. Additionally, the positive correlation observed between the immediate recall scores of the original RCFT and the coefficients of the shape similarity from our digital version also support the working memory deficit hypothesis.

One limitation of this study is the sample size. The relatively small sample size of the participants may restrict the validity and reliability of our results. However, to overcome this limitation, we enrolled participants who had undergone imaging and neuropsychological battery tests. The small sample size may also explain the lack of difference in visuoconstructional dysfunctions between patients with EOAD and patients with LOAD.

### Conclusions

We performed an analysis using a simplified RCFT that encompassed not only the quantification of the final figure but also the drawing process, such as pen strokes, spatial arrangement, and similarities, by using digital drawing data. Analyzing the drawing sequences of global and local elements as well as the number, length, and speed of strokes may only be possible through digital devices. Furthermore, our digitized version enabled automatic quantification of the degree of similarity and position of the drawing with respect to the target picture. Therefore, our digital metrics can complement the conventional visual rating of RCFT, which has been administered via pen and paper. Future standardization studies involving many patients and NCs are warranted to investigate whether our digitized, simplified RCFT would be useful in clinical and research settings.

## Appendix

Multimedia Appendix 1 Pseudocolored drawings for normal controls.

Multimedia Appendix 2 Pseudocolored drawings for patients with early-onset Alzheimer disease.

Multimedia Appendix 3 Pseudocolored drawings for patients with late-onset Alzheimer disease.

## Abbreviations

AD: Alzheimer disease
EOAD: early-onset Alzheimer disease
LOAD: late-onset Alzheimer disease
MCI: mild cognitive impairment
MMSE: Mini-Mental State Examination
MRI: magnetic resonance imaging
NC: normal control
RCFT: Rey-Osterrieth Complex Figure Test
SNSB: Seoul Neuropsychological Screening Battery

## References

[1] Cherrier MM, Mendez MF, Dave M, Perryman KM (1999). Performance on the Rey-Osterrieth complex figure test in Alzheimer disease and vascular dementia. Neuropsychiatry Neuropsychol Behav Neurol.

[2] Lezak M (2012). Neuropsychological Assessment. Fifth Edition.

[3] Waber DP, Holmes JM (1985). Assessing children's copy productions of the Rey-Osterrieth complex figure. J Clin Exp Neuropsychol.

[4] Somerville J, Tremont G, Stern RA (2000). The Boston qualitative scoring system as a measure of executive functioning in Rey-Osterrieth complex figure performance. J Clin Exp Neuropsychol.

[5] Nazar H, Moetesum M, Ehsan S, Siddiqi I, Khurshid K, Vincent N (2017). Classification of Graphomotor Impressions Using Convolutional Neural Networks: An Application to Automated Neuro-Psychological Screening Tests. Proceedings of the 14th IAPR International Conference on Document Analysis and Recognition.

[6] Müller S, Preische O, Heymann P, Elbing U, Laske C (2017). Increased diagnostic accuracy of digital vs conventional clock drawing test for discrimination of patients in the early course of Alzheimer's disease from cognitively healthy individuals. Front Aging Neurosci.

[7] Müller S, Herde L, Preische O, Zeller A, Heymann P, Robens S, Elbing U, Laske C (2019). Diagnostic value of digital clock drawing test in comparison with CERAD neuropsychological battery total score for discrimination of patients in the early course of Alzheimer's disease from healthy individuals. Sci Rep.

[8] Müller S, Preische O, Heymann P, Elbing U, Laske C (2017). Diagnostic value of a tablet-based drawing task for discrimination of patients in the early course of Alzheimer's disease from healthy individuals. J Alzheimers Dis.

[9] Canham RO, Smith SL, Tyrrell AM (2000). Automated Scoring of a Neuropsychological Test: The Rey Osterrieth Complex Figure. Proceedings of the 26th Euromicro Conference.

[10] Melrose RJ, Harwood D, Khoo T, Mandelkern M, Sultzer DL (2013). Association between cerebral metabolism and Rey-Osterrieth complex figure test performance in Alzheimer's disease. J Clin Exp Neuropsychol.

[11] Joubert S, Gour N, Guedj E, Didic M, Guériot C, Koric L, Ranjeva J, Felician O, Guye M, Ceccaldi M (2016). Early-onset and late-onset Alzheimer's disease are associated with distinct patterns of memory impairment. Cortex.

[12] Koedam EL, Lauffer V, van der Vlies AE, van der Flier WM, Scheltens P, Pijnenburg YA (2010). Early-versus late-onset Alzheimer's disease: more than age alone. J Alzheimers Dis.

[13] Smits LL, Pijnenburg YA, Koedam EL, van der Vlies AE, Reuling IE, Koene T, Teunissen CE, Scheltens P, van der Flier WM (2012). Early onset Alzheimer's disease is associated with a distinct neuropsychological profile. J Alzheimers Dis.

[14] Kang SH, Park YH, Lee D, Kim JP, Chin J, Ahn Y, Park SB, Kim HJ, Jang H, Jung YH, Kim J, Lee J, Kim J, Cheon BK, Hahn A, Lee H, Na DL, Kim YJ, Seo SW (2019). The cortical neuroanatomy related to specific neuropsychological deficits in Alzheimer's continuum. Dement Neurocogn Disord.

[15] Kang Y, Jahng S, Na DL (2012). Seoul Neuropsychological Screening Battery, Second Edition.

[16] Kang Y, Na D, Hahn S (1997). A validity study on the Korean Mini-Mental State Examination (K-MMSE) in dementia patients. J Korean Neurol Assoc.

[17] McKhann GM, Knopman DS, Chertkow H, Hyman BT, Jack CR, Kawas CH, Klunk WE, Koroshetz WJ, Manly JJ, Mayeux R, Mohs RC, Morris JC, Rossor MN, Scheltens P, Carrillo MC, Thies B, Weintraub S, Phelps CH (2011). The diagnosis of dementia due to Alzheimer's disease: recommendations from the national institute on aging-Alzheimer's association workgroups on diagnostic guidelines for Alzheimer's disease. Alzheimers Dement.

[18] Meyers J, Meyers K (1995). Rey Complex Figure Test and Recognition Trial.

[19] Lee BH, Chin J, Kang SJ, Kim E, Park KC, Na DL (2004). Mechanism of the closing-in phenomenon in a figure copying task in Alzheimer's disease patients. Neurocase.

[20] Piau A, Wild K, Mattek N, Kaye J (2019). Current state of digital biomarker technologies for real-life, home-based monitoring of cognitive function for mild cognitive impairment to mild Alzheimer disease and implications for clinical care: systematic review. J Med Internet Res.

[21] Casarotti A, Papagno C, Zarino B (2014). Modified Taylor complex figure: normative data from 290 adults. J Neuropsychol.

[22] de Paula JJ, Costa MV, de Andrade GD, Ávila RT, Malloy-Diniz LF (2016). Validity and reliability of a 'simplified' version of the Taylor complex figure test for the assessment of older adults with low formal education. Dement Neuropsychol.

[23] Wilson N, Batchelor J (2015). Examining Rey complex figure test organization in healthy adults. J Clin Exp Neuropsychol.

[24] Slavin MJ, Mattingley JB, Bradshaw JL, Storey E (2002). Local-global processing in Alzheimer's disease: an examination of interference, inhibition and priming. Neuropsychologia.

[25] Kwak YT (2004). 'Closing-in' phenomenon in Alzheimer's disease and subcortical vascular dementia. BMC Neurol.

[26] Chin J, Lee BH, Seo SW, Kim E, Suh MK, Kang SJ, Na DL (2005). The closing-in phenomenon in Alzheimer's disease and vascular dementia. J Clin Neurol.

[27] de Lucia N, Grossi D, Fasanaro AM, Carpi S, Trojano L (2013). Frontal defects contribute to the genesis of closing-in in Alzheimer's disease patients. J Int Neuropsychol Soc.

[28] Grossi D, de Lucia N, Trojano L (2015). Closing-in is related to apathy in Alzheimer's disease patients. J Alzheimers Dis.

[29] Serra L, Fadda L, Perri R, Caltagirone C, Carlesimo GA (2010). The closing-in phenomenon in the drawing performance of Alzheimer's disease patients: a compensation account. Cortex.

[30] Ambron E, Della Sala S (2017). A critical review of closing-in. Neuropsychology.

[31] van der Flier WM, Pijnenburg YA, Fox NC, Scheltens P (2011). Early-onset versus late-onset Alzheimer's disease: the case of the missing APOE ɛ4 allele. Lancet Neurol.

[32] Bauer RM, Parsons MW, Hammeke TA (2014). Visuospatial, visuoperceptual, visuoconstructional disorders. Clinical Neuropsychology: A Pocket Handbook for Assessment.

[33] Stevens A, Bernier R, Volkmar FR (2013). Visual-motor function. Encyclopedia of Autism Spectrum Disorders.

