# Supplementary figures and images for: A Comprehensive Evaluation of the Process of Copying a Complex Figure in Early- and Late-Onset Alzheimer Disease: A Quantitative Analysis of Digital Pen Data
Source: J Med Internet Res. 2020 Aug 12;22(8):e18136. doi: 10.2196/18136 (PMC7450382; doi:10.2196/18136)

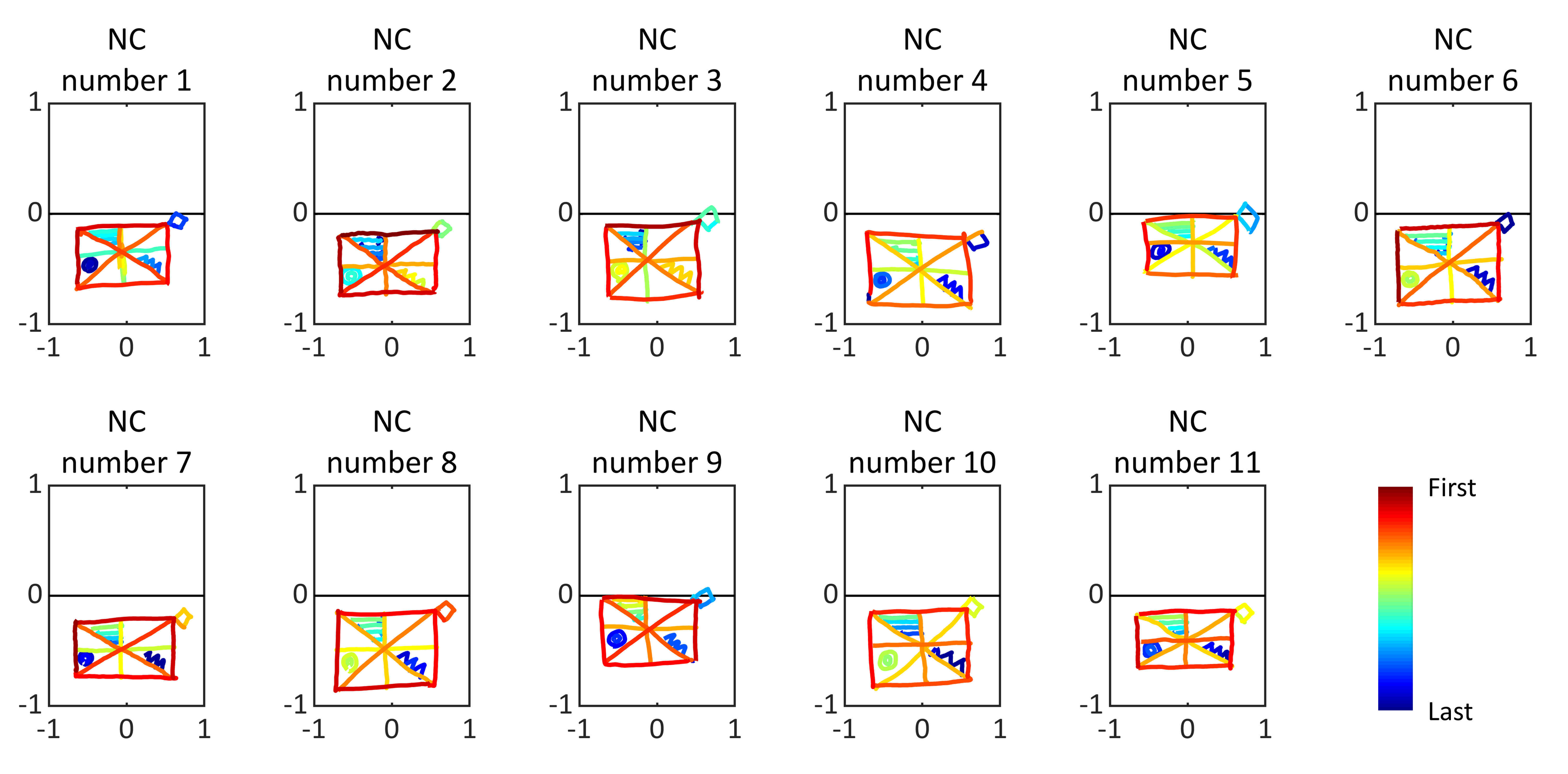

Supplement: Multimedia Appendix 1 [file jmir_v22i8e18136_app1.png]

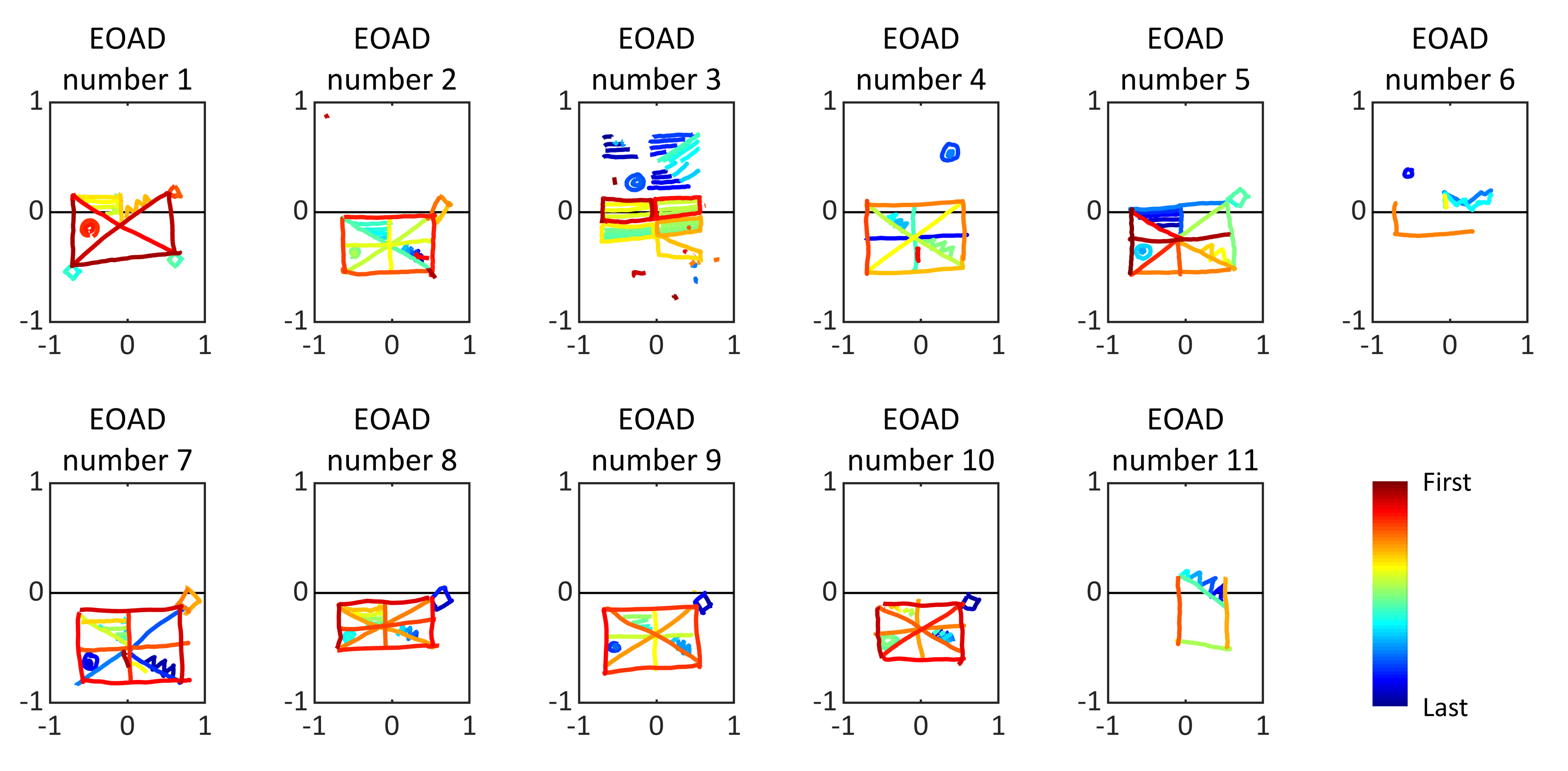

Supplement: Multimedia Appendix 2 [file jmir_v22i8e18136_app2.png]

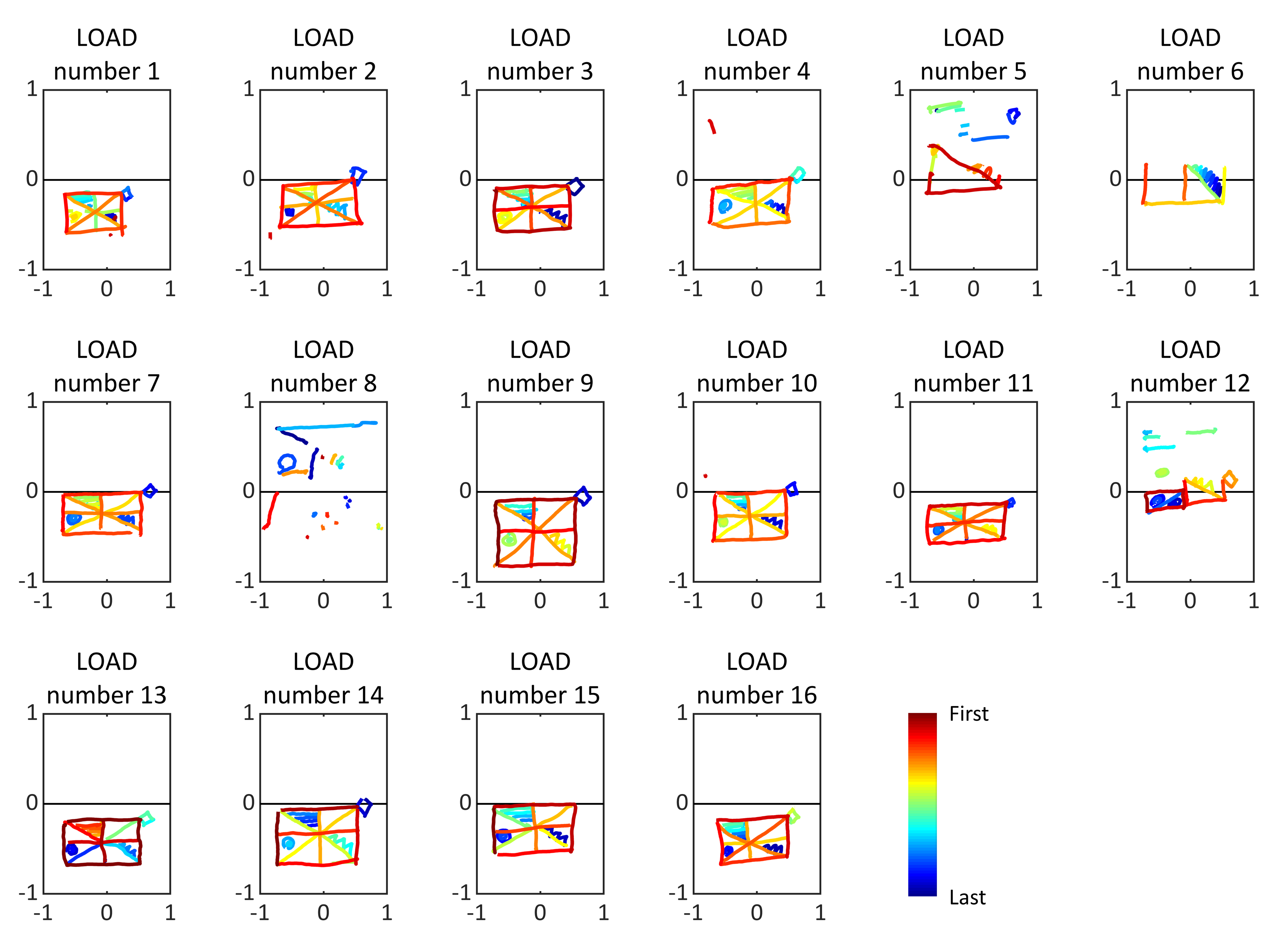

Supplement: Multimedia Appendix 3 [file jmir_v22i8e18136_app3.png]
